# Undergraduate research in medicine: A summary of the evidence on problems, solutions and outcomes

**DOI:** 10.1016/j.amsu.2022.103280

**Published:** 2022-01-25

**Authors:** Laura Marcela Mass-Hernández, Laura Marcela Acevedo-Aguilar, Ivan David Lozada-Martínez, Lucas Santiago Osorio-Agudelo, Juan Gabriel Esteban Maria Maya-Betancourth, Omar Andrés Paz-Echeverry, Mario Javier Paz-Echeverry, Harold Sebastian Castillo-Pastuzan, Juan Carlos Rojas-Pimentel, Sabrina Rahman

**Affiliations:** a Medical and Surgical Research Center, Future Surgeons Chapter, Colombian Surgery Association, Cartagena, Colombia; b Grupo Prometheus y Biomedicina Aplicada a las Ciencias Clinicas, School of Medicine, Universidad de Cartagena, Cartagena, Colombia; c School of Medicine, Universidad CES, Medellin, Colombia; d Grupo de Interés en Dermatología y Medicina Interna (DERMIUTP), School of Medicine, Universidad Tecnológica de Pereira, Pereira, Colombia; e School of Medicine, Universidad del Cauca, Popayán, Colombia; f School of Medicine, Universidad de Nariño, Pasto, Colombia; g School of Medicine, Universidad de Santander, Bucaramanga, Colombia; h Independent University, Dhaka, Bangladesh

**Keywords:** Research, Undergraduate medical education, Evidence-based medicine, Curriculum

## Abstract

Scientific research plays a fundamental role in current medical practice and it is of great importance that medical students relate to it from the beginning of their professional career, since it generates multiple benefits that will be reflected during the course of their careers as university students and future professionals. However, getting involved in research during the undergraduate years is not always easy, as there are different obstacles and challenges that result in a reduced number of research students. Because of this, it is necessary to adopt appropriate strategies and measures to help facilitate this process, in order to foster the early development of essential skills that will facilitate responsible clinical practice. Knowing the evidence on this issue is fundamental to propose educational solutions depending on each context.

## Highlights

• Undergraduate research is the best way to promote the curriculum.
• Undergraduate research increases the likelihood of publishing articles of higher quality.
• Undergraduate research increases the likelihood of publishing more articles.

## 1 Introduction

In the continuing quest for safe and effective care for global health needs, evidence-based medicine remains the cornerstone of clinical practice decision making. Ensuring the attainment of knowledge and skills of the student and general practitioner is the most important task to maintain quality standards and reduce malpractice errors. It has been shown that participation in research and scientific publication from undergraduate level, specifically in the training of data analysis, problem-posing questions, critical reading of the literature and scientific writing, contribute substantially to the development of the minimum necessary skills [1,2].

But the importance of scientific research for physicians goes beyond the evolution of their scientific skills. Those students who do research are more likely to republish scientific articles, publish articles of better quality, have professional satisfaction, and have better economic and academic stability [3]. In this order of ideas, it is imperative that all medical students understand and get involved in a committed manner in the field of research and thus, as a future professional, manage to keep themselves always updated and supported by evidence of the best quality [4].

Despite the overwhelming importance of medical research for society, there is a marked difference in scientific productivity between high-income and low- and middle-income countries, based on the low investment in research by the governments of each country; according to 2010 statistics, the U.S. had 3867 researchers per million people, as opposed to Colombia and Venezuela, which have 193 and 200 researchers per million people, respectively [5]. But there is also a macro component that is difficult to counteract by research, and that is the underestimation of its validity by society itself; a similar aspect experienced by students on the part of professors and senior researchers.

In general terms, surveys have found that medical students have a very positive outlook on scientific research, but they face multiple barriers that limit their participation in the approach and development of scientific projects, so they do not conceive of ever assisting in a scientific study [6,7]. However, economic and social interests have a negative influence on the development of a research career, since it is not well paid in low- and middle-income countries. This, together with the unfavorable working conditions of physicians in the Third World, constitutes a false impasse [8]. In order to justify the need to seriously involve the research component in the physician, which is mainly focused on the clinical part, it is necessary to know the problems, possible solutions and outcomes of research during undergraduate studies [9,10]. Therefore, the aim of this review is to summarize the relevance of research during undergraduate medical school, the problems, solutions and outcomes reported in the literature, and to discuss new horizons to provide a real solution to the limitations of medical student participation in research.

### 1.1 Research in the medical career: when and why?

The main reason for researching and publishing high quality scientific articles lies in learning to use and critique evidence for responsible decision making based on it, trying to avoid as much as possible medical failures during clinical practice [11,12]. On the other hand, this tool helps to optimize overall critical thinking, personal satisfaction and deploy the academic experience enriching the *curriculum*, which is fundamental when it comes to gaining entry into competitive residencies and other types of graduate programs [13,14]. A study conducted in 2016 that sought to investigate the reasons why medical students decided to research and publish scientific articles, yielded as the main response "to increase competitiveness to apply to residency", mainly in those specialties with high concurrence, such as surgical residencies [15]. However, beyond access to residencies, it has been proven that participation in scientific research during undergraduate studies leads to a greater probability of obtaining a master's or doctoral degree, even with a better academic performance and in less time, due to the development of excellent leadership skills, teamwork, communication, among many others that come with collaborative work in science [16].

But, when is the best time to start research? After having analyzed numerous studies, it has been concluded that it would be ideal to encourage research from the beginning of the medical career [17], since it has been proven that those undergraduate students who research and publish more manuscripts before graduating, tend to continue publishing after obtaining their medical degree and even manage to publish more manuscripts (up to 1.7 more times) compared to those who start publishing after graduating [9,10]. In addition, knowing basic concepts related to evidence-based medicine and scientific communication and publication facilitates the search for information during the study for the academic curriculum.

### 1.2 Long-standing problems of student medical research

Although the role of undergraduate research in medicine has always been highlighted, over the last two decades the participation of medical students in this field has been limited by several obstacles, the main one being the lack of stimulation and scientific training by medical schools, which leads to a reduced number of research students [9,18]. In a study published in 1995, conducted by the Stanford University, a study was conducted to evaluate the incentive that the university gives to its students to participate in research, where recent graduates were asked about the value of their undergraduate research experience, and it was found that 90% of them had participated in scientific projects, 75% had managed to publish at least one article and 67.5% revealed that the experience motivated them to continue their research [19]. Likewise, in a study published in 2004 by the University of Queens, in an attempt to encourage students to participate in scientific projects and to pursue research careers, they included a mandatory "Critical Research" elective in the curriculum during their second year of study, showing a considerable increase in the number of students who expressed an interest in pursuing a research career after completing the elective [20]. The above suggests that probably the abandonment of participation in research is due to the inability to understand complex analysis and issues of scientific publication, which are not really part of the objective of knowing the basics of evidence in medicine and initiation in research, but of the work done by experts with high level academic preparation; that is, it is necessary that the student understands and learns what is according to their level, and progressively become involved in more complex issues. This can be clearly stimulated by the undergraduate academic curriculum.

Other of the main long-standing barriers to scientific research and publication were reflected in a study by Remes et al. [21], where after evaluating the research participation of their students, it was found that there were unequal opportunities between men and women (as evidenced by a higher percentage of articles published by men) and the deficiency, both qualitative and quantitative, of tutors to adequately supervise the performance of the students [21]. Actually, it can be perceived that the barriers that have affected the participation of medical students in research for decades are multiple, and encompass fields that go beyond research itself. This is probably the reason why many of these difficulties persist today.

### 1.3 Current challenges in student medical research

According to the above scenarios, several studies have shown the importance of student support from their school, where teachers are a key guide for students to awaken their interest and commitment to research from the beginning of their careers, in order to combat the absence of research students [22]. However, currently this number of research students is still quite low, due to a deficit in basic scientific training and motivation on the part of medical schools [23], in addition to other limiting agents such as the lack of time of the medical student due to the academic load, difficulty in choosing a specific topic to work on, lack of tutors or professors with experience in conducting research, among others [24,25]. Likewise, the long-standing problem raised in the previous section of this review, regarding the inequality of opportunities between men and women, is still relevant today in the research field, because although the number of women researchers has been increasing over time, there are still a greater number of articles published by men [26].

On the other hand, another challenge that arises in this context is that nowadays there is a preference for manuscripts prepared by professionals or specialists, a situation known as "scientific racism", where the work and dedication of undergraduate students is underestimated [27]. This type of racism is also reflected in the preference for scientific papers written by native authors from developed countries, where it is presumed that all papers from this region are of high quality, and where advantage is taken of the fact that the few journals with high impact, strong funding and use of the universal language in science (English), come from first world countries [28]. And because the materialization of scientific production, competition and the academic and job market demand products published in high impact journals, the difficulty of achieving this titanic accomplishment substantially decreases the interest in persisting in research.

Furthermore, it is clear that one of the main motivations for medical students to engage in scientific research is the enrichment of their curriculum vitae to gain entry into a graduate program, which on the one hand is highly beneficial for the student's professional career, but on the other hand, drives students to prioritize quantity over quality of scientific manuscripts, taking into account the time limit during their undergraduate studies and the job market, reducing the probability of conducting prospective studies or high-impact publications [15].

### 1.4 Solutions to facilitate research during a medical career

Research plays an important role in scientific advancement and is crucial in strengthening medical learning and practice [24]. Because of this, it is essential to inculcate and promote the development of critical and rational thinking among medical students from the beginning of their professional career, in order to obtain better training in the area of research and evidence-based medicine [29]. However, in order to overcome the many challenges that persist today and to finally achieve active student participation in research, it is necessary to develop appropriate strategies and measures to help facilitate this process [30].

One of the most important aspects of adopting a research culture during undergraduate medical school is to create a curriculum that educates students from the beginning of their careers about the basic principles and parameters of research, i.e., epidemiology, biostatistics, research methodology, and scientific publication [17]. To this end, one of the strategies that can facilitate this objective is the creation of interest groups, which consist of academic and research communities that seek to provide meaningful training to medical students in different areas of knowledge and thereby create spaces that promote the dissemination of scientific knowledge [24,31]. These interest groups can be formed by undergraduate students, residents and professors who fulfill the role of mentors as experts in certain topics and who act as guides to carry out projects and participate in events of social appropriation and circulation of knowledge [32,33]. Currently, there is evidence available that supports the importance of the integration of these groups in the different medical schools, which through various activities, workshops, seminars and research projects, help to form more comprehensive professionals by being closely related to evidence-based medicine, in addition to being able to contribute to the production of scientific knowledge in medicine [34].

On the other hand, a strategy that could be useful to combat the obstacles related to the rejection of research papers due to the educational level of the authors would be to create sections in high impact journals reserved especially for the publication of papers developed by medical students [24]. It is also extremely important to develop strategies to promote international collaboration with researchers of great trajectory and experience in order to improve the academic quality of students and thus provide more tools to produce studies of increasingly higher quality [35]. Finally, it is necessary to provide research scholarships during the course of the degree, which offer the opportunity to demonstrate the research potential of students and encourage internships at research institutes [36].

## 2 Outcomes of undergraduate medical research

Participation in research during undergraduate medical school is associated with the acquisition of greater research-related competencies, improved clinical outcomes in the context of health care, and greater scientific productivity in the future (Table 1) [[37], [38], [39], [40], [41], [42]]. In addition, it is probably the best option to improve your resume in a short period of time when applying for a medical residency or other postgraduate degree [39].

**Table 1:** Summary of some studies that have evaluated undergraduate medical research outcomes [[37], [38], [39], [40], [41], [42]].

Authors	Objective	Methods	Outcomes	Conclusions
Al-Busaidi et al. [37]	To assess the effects of scientific publication in the New Zealand Medical Student Journal (NZMSJ) by undergraduate students	Papers by medical students published in the NZMSJ between 2004 and 2011 were identified and retrospectively analyzed	NZMSJ student authors published more articles before graduation compared to controls (p = 0.01). Such behavior persisted after graduation (p < 0.001)	Publishing as an undergraduate student in a medical student scientific journal correlates with higher number of publications during and after undergraduate studies
Sorial et al. [38]	Determine the effect on medical career enhancement and academic benefits of research intercalation	Retrospective electronic survey-type study of students who undertook research internships between 2005 and 2012	87% of the participants advanced through their graduate training in the minimum amount of time. Additionally, more than 73% actively continued with research after graduation	The research intercalation evidenced a beneficial impact on postgraduate medical career advancement and contributed to participants' continued development of high quality research
Weaver et al. [39]	Investigating the long-term outcomes of mandatory and elective research during undergraduate study in a U.S. medical school	Retrospective qualitative study	Performing research during undergraduate studies was a positive predictor for persisting with research during specialized academic training (OR 27.42; 95% CI, 2.77–284.32; p = 0.005). Additionally, it was also favorably related to employment opportunity in academic medical centers (OR 4.82; 95% CI, 1.49–15.65; p = 0.009)	A positive association was identified between undergraduate research and current research output
Waaijer et al. [40]	To assess whether students who publish during their undergraduate career have higher scientific productivity after graduation compared to those who do not publish during their medical career	Bibliometric study	The estimate showed that publishing during undergraduate studies increases the probability of publishing after graduation by up to twice as much (RR: 1.90; CI 95%, 1.76–2.05; p < 0.001)	Students who published during their undergraduate studies have a higher number of publications after graduation compared to those who did not publish during their undergraduate studies
Ommering et al. [41]	To examine whether an achievement in a required research course is associated with increased motivation with respect to the research area	Prospective cohort study	Achievement in oral presentation (β = 0.115; 95% CI, 0.017–0.214; p = 0.022), and research report (β = 0.114, 95% CI, 0.017–0.0211; p = 0.022) were found to be associated with higher intrinsic motivation with respect to the research area	Obtaining achievement in a required research course by writing a research report and making an oral presentation along the same lines increases intrinsic motivation in undergraduate students for research
Ommering et al. [42]	To evaluate whether the motivation to do research is the basic step to promote the training of future physician-scientists	Prospective cohort study	It was found that first year students who presented higher scores in terms of intrinsic motivation to do research were more related to research in their second year (OR 3.4; 95% CI, 2.08–5.61)	Intrinsic motivation to do research during the early undergraduate years could be the foundation step for encouraging future research participation

For their part, medical students themselves demonstrate a positive attitude and perception of the importance of early medical research as a tool of great value when applying for specialized training and also consider it very useful for becoming more comprehensive and skilled future professionals [[38], [39], [40], [41], [42], [43], [44]]. A study conducted in Saudi Arabia to assess students' attitudes towards research participation during their medical career showed that 58.1% of the respondents were aware of the significant relevance of being involved in research during the course of their medical training, which supports the positive mindset expressed above [45]. In other words, the relevance of research in medicine is a fact among medical students; it is only necessary to incorporate the skills and help them to materialize the production where they participate.

### 2.1 Effects of student research on global medicine

Research in undergraduate students is essential to achieve great advances in clinical medicine, since by increasing their early training in research areas, favorable results are obtained in the context of their health practice, thus allowing them to generate answers to global health problems in a safer and more effective way [46,47]. Because of this, it is very important to invest in education and training of researchers in order to build a healthier community in the long term based on the great impact they represent in global medicine [44]. This statement can be supported by the existing evidence about the role played by some medical students in extremely important aspects in the clinical area throughout history, as is the case of Charles Best, who contributed to the discovery of insulin, Paul Langerhans, who is related to the discovery of the islets of Langerhans, being these discoveries made during his undergraduate studies [48].

### 2.2 Is a radical change in the medical curriculum necessary?

As evidenced throughout the literature review, participation in research areas offers important skills in a number of spheres [49]. Therefore, it is necessary to analyze and modify, if necessary, the curricula of medical schools, so that they include the practice of research in order to create awareness in students about the usefulness of research and to develop early on fundamental attitudes in them for the development of an adequate medical profession [50,51]. In addition, for students it also seems advantageous, in terms of motivation, to encourage situations in which they can experience autonomy and independent work, which leads to the need to opt for more active training approaches during education [52]. This is supported by studies indicating that medical schools that have included mandatory research courses in their curriculum have shown that more students, upon graduation, show a greater interest in research [53]. Thus, it is clear that during the course of undergraduate studies, the basic principles of the scientific method, the different methods of medical research, the use of evidence-based medicine in clinical practice, and even more advanced research elements that can be trained through activities such as conferences, workshops, research interest groups, among others, should be incorporated into the curriculum [54]. Finally, it should be noted that in order to achieve the desired objectives, students need to have adequate collaboration and guidance from experienced researchers who closely follow the steps taken by each of them in the context of the research, which should also be taken into account when making adjustments in the curricula of medical schools around the world [52].

The change in the curriculum of medical schools with the incorporation of the research component would have a much greater impact than expected from the simple production of the medical student alone [[55], [56], [57]]. As the demand for scientific interest and participation in general increases, it will be necessary for schools and governments to invest in the purchase of equipment, databases and software that facilitate the conduct of research, data analysis and scientific publication [58,59]. This will help the massive creation of medical interest groups, which in turn, through collaborative work, will reinforce aspects of professionalism in medicine from undergraduate level, reducing the frequency of negative events such as harassment and discrimination [60,61]. The massive participation of students will facilitate the direction of a new line of professionals with medical training who will provide answers from translational medicine to clinical questions [62,63]. Probably the potential for scientific development of a third world country is found in its undergraduate students, and in medicine, there are many projects that remain to be carried out. In spite of the existing avalanche of literature on medical education, the problems of research during undergraduate medical education are still valid [[61], [62], [63]].

## 3 Conclusions

Research and publication of scientific projects during undergraduate studies is a fundamental pillar that all medical students should learn to develop from the beginning of their careers, since beyond the enrichment of their resumes as future physicians. This training provides the development of substantial skills and competencies that allow them to respond in the most effective way to the needs of global health. Considering that those students who research and publish during their undergraduate studies tend to publish more and better quality articles after graduation, it is necessary to reinforce this interest in order to encourage a culture of high quality research.

## Ethical approval

It is not necessary

## Sources of funding

None.

## Author contributions

All authors equally contributed to the analysis and writing of the manuscript.

## Trial registry number

1. Name of the registry: Not applicable.
2. Unique Identifying number or registration ID: Not applicable.
3. Hyperlink to your specific registration (must be publicly accessible and will be checked): Not applicable.

## Guarantor

Sabrina Rahman. Independent University, Dhaka, Bangladesh. sabrinaemz25@gmail.com

## Declaration of competing interest

The authors declare that they have no known competing financial interests or personal relationships that could have appeared to influence the work reported in this paper.
